# Inquiring into the Gaps of *Campylobacter* Surveillance Methods

**DOI:** 10.3390/vetsci4030036

**Published:** 2017-07-19

**Authors:** Maria Magana, Stylianos Chatzipanagiotou, Angeliki R. Burriel, Anastasios Ioannidis

**Affiliations:** 1 Department of Biopathology and Clinical Microbiology, Aeginition Hospital, Athens Medical School, Athens 15772, Greece; mariamgn91@gmail.com (M.M.); schatzi@med.uoa.gr (S.C.); 2 Department of Nursing, Faculty of Human Movement and Quality of Life Sciences, University of Peloponnese, Sparta 23100, Greece; aburriel@uop.gr

**Keywords:** Campylobacteriosis, methodology, molecular typing, human infection, zoonosis, surveillance, epidemiology

## Abstract

*Campylobacter* is one of the most common pathogen-related causes of diarrheal illnesses globally and has been recognized as a significant factor of human disease for more than three decades. Molecular typing techniques and their combinations have allowed for species identification among members of the *Campylobacter* genus with good resolution, but the same tools usually fail to proceed to subtyping of closely related species due to high sequence similarity. This problem is exacerbated by the demanding conditions for isolation and detection from the human, animal or water samples as well as due to the difficulties during laboratory maintenance and long-term storage of the isolates. In an effort to define the ideal typing tool, we underline the strengths and limitations of the typing methodologies currently used to map the broad epidemiologic profile of campylobacteriosis in public health and outbreak investigations. The application of both the old and the new molecular typing tools is discussed and an indirect comparison is presented among the preferred techniques used in current research methodology.

## 1. Introduction

*Campylobacter* is one of the most common pathogen-related causes in diarrheal illnesses globally and has been recognized as a significant factor of human disease for over three decades [1]. Campylobacteriosis is a self-limiting infection with enteritis, abdominal cramps, fever, nausea and vomiting as the main manifestations. Besides the gastrointestinal symptoms of *Campylobacter* infection, the extra-gastrointestinal manifestations include cases of reactive arthritis, septicemia, endocarditis, meningitis, brain abscesses, bone and soft-tissue infections, periodontitis and the Guillain–Barré and Miller Fisher neurological syndromes [2]. Due to the self-limiting character of the disease, most campylobacteriosis cases simply require supportive therapy including hydration and maintenance of electrolytes balance [3]. Antibiotic therapy is indicated only in severe and persisting infections in sensitive populations including children, the elderly, pregnant women and immunocompromised patients, as well as in cases of extra-gastrointestinal manifestations. Ciprofloxacin is used for the empirical treatment of travel-related gastroenteritis but macrolides are the treatment of choice [4].

The majority of campylobacteriosis cases go undiagnosed or under-reported due to the self-limiting character of the disease. However, according to the Foodborne Diseases Active Surveillance Network (FoodNet), 14 cases of campylobacteriosis are diagnosed per 100,000 population in the United States (U.S.) (approximately 1.3 million persons) and 71 cases per 100,000 population in the European Union (EU) (approximately 200,000 persons) annually [5,6]. Campylobacteriosis is rarely a fatal disease, and rare mortality reports are usually confined to extreme age groups and/or immunocompromised patients [7]. It has been estimated that approximately 76 persons in the US with *Campylobacter* infection die annually, while in the EU the reported deaths in 2015 accounted for 59 in a total number of 229,213 human cases [5,6].

For years, *Salmonella* was the number one cause of enteric infections within the EU representing a significant challenge to public health; however, the scenery has changed since the increased trend of *Campylobacter* spp. infections [8,9]. According to recent data from the European Food Safety Authority (EFSA) and the European Centre for Disease Prevention and Control (ECDC), in terms of zoonoses and foodborne outbreaks, human campylobacteriosis is the most commonly detected zoonosis in the EU exceeding salmonellosis cases [9]. Most animals serve as reservoirs of *Campylobacter* species and only a small number is afflicted by campylobacteriosis [10]. In fact, a decreasing rate of campylobacteriosis cases is reported in animals compared to 2014 in the EU, due to an overall lack of surveillance data. In general, spatiotemporal comparisons of campylobacteriosis incidence rates in various animals among the EU countries are difficult; variations in data acquisition stem from inconsistent sampling procedures and testing methodologies [6].

The diverse members of the *Campylobacter* genus, most commonly represented by *Campylobacter jejuni* and *Campylobacter coli* in both humans and animals, constitute a large number of either unknown or newly identified species. Among the *Campylobacter* species, *Campylobacter jejuni* subsp. *jejuni* is most frequently isolated in human gastroenteritis accounting for approximately 90% of campylobacteriosis cases, followed by *C. coli* [11,12,13]. According to previous reports, the *Campylobacter* genus consists of 16 species and six subspecies, while the total species number has been rearranged to 36 species including both the “emerging” human and animal pathogens (*Campylobacter upsaliensis*, *Campylobacter hyointestinalis*, *Campylobacter ureolyticus*, *Campylobacter concisus*, *Campylobacter lari*, *Campylobacter fetus*) and the novel *Campylobacter hepaticus* sp. nov. [2,14,15].

However, it is of great significance that there has been a failure of phenotypic markers to differentiate isolates at family and genus level, which has historically changed the “map” of the order of Campylobacterales. The exclusion of several species from the *Campylobacter* genus and their new taxonomy into different genera, according to the distance among species in phylogenetic analyses, has gone silent in the past two decades [16]. Namely, *Campylobacter pyloris, Campylobacter cinaedi* and *Campylobacter fennelliae* are now transferred to the *Helicobacter* genus (family of Helicobacteraceae), while *Campylobacter butzleri, Campylobacter nitrofigilis,* and *Campylobacter cryaerophila* are now members of the *Arcobacter* genus (family of Campylobacteraceae) [16,17,18,19].

Despite the confusing status regarding the true number of *Campylobacter* species, molecular techniques and their combinations have allowed for species identification among members of the *Campylobacter* genus at a rather increased resolution rate, but the same tools usually fail to proceed to subtyping of closely related species due to high sequence similarity [20]. Typing and subtyping failure does not apply for *C. jejuni* and *C. coli* which are the most popular campylobacters in the research milieu; numerous studies involve the two pathogens and this fact could probably stem from the fact that other clinically significant pathogens have been transferred from the *Campylobacter* genus to another genus as previously discussed. Additionally, there is a gap in determining the link between human infection and the source of infection. The pathogenicity and clinical relevance of the emerged campylobacters are still unidentified and the interrelationship of the environmental reservoirs with the human host remains ambiguous [11].

In an effort to shed light on the above-mentioned inquiries, this review aims to (i) discuss the absence of ideal storage conditions of campylobacters that could facilitate a more comprehensive sample analysis; (ii) critically revisit the inadequacies of detection/identification methods used in laboratory routine; and (iii) underline the strengths and limitations of currently used molecular typing methodologies in mapping the broad epidemiologic profile of campylobacteriosis.

## 2. Gaps in *Campylobacter* spp. Identification

The argument that we still have no proper appreciation of the relative importance of the emerging species hampers further development of subtyping methods. Greater focus should be placed on closer detection of the emerging species and improved microbiological methods for enhanced cell recovery from clinical and environmental samples. Determination of the relative prevalence of these species in clinical specimens will provide answers regarding the necessity for subtyping methods development for epidemiological investigations.

The first observations suggesting that *Campylobacter*-like isolates are potential human pathogens associated with gastrointestinal infections in both healthy and immunocompromised hosts stems back to the 1980s [21]. Since then, a multitude of novel *Campylobacter* species has emerged, linked with campylobacteriosis manifestations, colonizing a diverse number of niches in human. Reports implicate *C. concisus*, *Campylobacter curvus*, *C. fetus* subsp*. fetus*, *Campylobacter gracilis*, *Campylobacter helveticus*, *Campylobacter hominis*, *C. hyointestinalis*, *C. insulaenigrae*, *C. lari*, *Campylobacter lanienae*, *Campylobacter peloridis*, *Campylobacter mucosalis*, *Campylobacter showae*, *Campylobacter sputorum* biovar *paraureolyticus*, *C. upsaliensis* and *C. ureolyticus* in diarrhea and vomiting [22,23,24,25,26,27,28,29,30,31,32,33], and most of them have been recovered from blood samples of bacteremic patients [24,28,34,35,36,37]. There are also case reports of hospitalized humans due to life-threatening complications by *Campylobacter*-related species (namely *C. concisus*, *C. curvus*, *C. fetus* subsp*. fetus*, *C. gracilis*, *C. rectus*, *C. peloridis*, *C. showae*, *C. sputorum* biovar *sputorum*, *C. upsaliensis* and *C. ureolyticus*) isolated from the cerebrospinal and peritoneal fluid, the axillary nerve, hepatic, lung, genitalia and brain abscesses as well as from soft tissue lesions, bone infections and thoracic empyema [28,38,39,40,41,42,43,44,45,46,47]. In animals, the species *C. avium* has been isolated from the cecal contents of chickens and turkeys, *C. canadensis* from the cloacal swabs of whooping crane, *Campylobacter cuniculorum* from the cecal contents of rabbits, *C. subantarcticus* from the fecal swabs of albatross chicks and gentoo penguins, *Campylobacter troglodytis* from the stools of chimpanzees, and *Campylobacter volucris* from the cloacal swabs of gulls [48,49,50,51,52,53]. In humans, there is one case reporting bacteremia associated with *C. volucris* in a cirrhotic patient with polycythemia vera and one case of *C. troglodytis* isolated from infants’ diarrheic stool samples in Tanzania, Bangladesh, and Peru [54,55]. All other *Campylobacter* species found in human are also isolated mostly from the feces of domestic and wild animals implying the fecal-oral route of transmission; however, the complete mechanisms of the human host infection are not completely understood.

The similarity in the isolated species found both in humans and in animals indicate that the environment, including food and water products, plays a significant role in the transmission of emerging *Campylobacter* species. However, their isolation and identification are not easy and always successful procedures. Robust assays targeting features conserved in each species and that can be used to differentiate it from other species would improve the procedure of identification of the various campylobacters [56,57]. But why do emerging *Campylobacter* spp. detection and identification fail? Apart from the protocols for the laboratory growth and isolation of the fastidious *Campylobacter* spp. that may not be routinely followed (hydrogen-enriched atmospheric conditions, antibiotic-enriched culture media, incubation for up to 7 days with close monitoring of growth), the contamination from non-fastidious microorganisms, the delayed specimens handling, as well as the isolates loss during extensive freeze-thaw cycles and suboptimal storage of the bacterial samples set inevitable risk factors for detection and identification failure. The isolates loss is a “silent risk” that affects the survival and the identification at species and strain-level, and has raised concerns to the scientific community, creating the need for optimal storage and maintenance conditions. The thermophilic *Campylobacter* is particularly sensitive in temperature changes, thus extensive freeze—thaw procedures lead to reduction of the population of *Campylobacter* spp., entrance in the viable but non-culturable (VBNC) state, and potential loss of novel species and/or strains [14,58].

For years, the *Campylobacter* storage has been a hot issue and several protocols have emerged. Additionally, the existence of the “protective shield” of a multispecies biofilm community could hide a wide array of emerging *Campylobacter* species, while the metabolically inactive persister cells which can effectively “escape” adverse environments and regain the ability to cause infection when found in optimal circumstances may also lead the identification process to erroneous results [11,59]. Finally, the VBNC campylobacters that retain their virulence and physiology—but cannot be cultured in standard culture media—are highly resistant to external stresses such as pasteurization, and their presence in food sets a serious challenge for public health [60,61,62].

Laboratory diagnosis of campylobacteriosis caused by species other than *C. jejuni* and *C. coli* is complicated due to the demanding growth and identification procedures of the various subsets of species. Both the culture-dependent (biochemical tests) and culture-independent (PCR-polymerase chain reaction, immunological assays) methodologies present inconsistent and suboptimal data regarding sensitivity, providing evidence that there is not a single gold standard method for *Campylobacter* identification, but the preferred path is the combinatorial application of the available molecular methods [2]. Traditional culture-dependent methods based on colony appearance on charcoal cefoperazone deoxycholate agar (CCDA) or other *Campylobacter-*specific media in the presence of antibiotics, microaerobically incubated at 41.5 °C for 48 h, followed by typical biochemical testing (oxidase/catalase tests, hippurate hydrolysis) in pure cultures, often fail to properly identify pathogens at species and strain level [63,64,65]. Culture-dependent methods serve in the identification of phenotypic traits but fail to overcome the burden of high sequence similarity of the *Campylobacter* species.

Culture-independent methods include molecular identification by using nucleic acid amplification tests (NAATs), offering enhanced sensitivity in the determination of the bacterial genetic traits [2,65]. PCR amplification of the 16S rRNA gene is a popular tool for *Campylobacter* detection; however, PCR is a labor-intensive and time-consuming methodology and the fact that the 16S rRNA gene needs species-specific primers fails to differentiate closely related *Campylobacter* species [66,67,68]. A costly yet reliable solution to this problem is the construction of a phylogenetic tree by combining the 16S and 23S rRNA genes with the internal transcribed spacer (ITS) region, offering a high-resolution differentiation at a species and strain level [31]. Another culture-independent method is protein composition analysis of the bacterial cell by using the principle of the matrix-assisted laser desorption ionization time of flight mass spectrometry (MALDI-TOF MS). This method offers pure bacteria culture identification at the species-level in a time-efficient manner, requires less effort than the DNA-based methods, is cost-effective and provides reproducible results with high sensitivity [69]. Another key property of this method is its ability to identify multiple members of the *Campylobacter* genus in mixed cultures [70]. MALDI-TOF MS has been applied for species-level identification for a wide array of campylobacters as they are the well-known *C. jejuni, C. coli,* as well as for the emerging *C. lari, C. fetus, C. hyointestinalis, C. upsaliensis, and C. sputorum* [70,71]. Novel taxa differentiation at the subspecies level in the emerging campylobacters group would be difficult by applying conventional phenotypic tests. However, MALDI-TOF MS enables such a discrimination through phenotypic biomarkers [72].

## 3. Molecular Typing Tools: Getting to Know Each Other

Molecular typing tools (i) are widely applied in the identification of novel bacterial strains, (ii) aid the discrimination of closely related isolates, (iii) aim at the study of the bacterial organization at the genome level, and (iv) track infection patterns and routes of transmission [57,73]. Molecular methodologies for the differentiation of *Campylobacter* at species and strain level have overcome the burdens of traditional phenotype-based techniques and have enhanced the discrimination power in epidemiology surveillance and outbreak detection; nevertheless, the reported cases of *Campylobacter* infections to date reflect only partially the actual magnitude of the disease [56,74]. Accuracy is the number one factor in strain differentiation and identification, therefore, careful processing of data is a necessity for molecular epidemiology regarding both human infections and environmental surveys. In general, high quality typeability is mandatory for all typing methods, yet the choice of the ideal method should be based on the epidemiological and spatiotemporal context to be applied and should incorporate several features in order to meet specific practical requirements [75,76].

In the clinical setting as well as in research laboratories, the implementation of molecular typing methods requires rapid and easy-to-perform analysis. Another significant characteristic of the ideal typing method should be deployability, therefore offering high-throughput techniques by using standard and inexpensive laboratory equipment [56,76]. The validation of the tools applied for molecular typing must meet performance criteria referring to the stability, reproducibility and the portability of the analysis [57]. Molecular typing tools allow accessibility from electronic databases offering surveillance at a larger scale. Improved bioinformatics algorithms for data mining and sharing have made it possible for *Campylobacter* typing networks to universally communicate the results of phylogenetic analyses.

**Figure 1:**
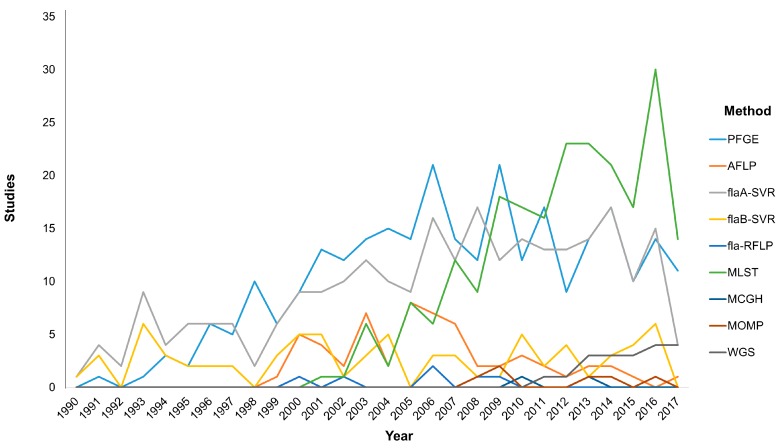
Graphical analysis of the applied molecular typing methodologies over time based on PubMed search. The use of molecular methodologies for the differentiation of *Campylobacter* at species and strain level has been modified during the last decades according to the new trends in technology, the improvements in bioinformatics, and the needs of the scientific community. Pulsed-field gel electrophoresis (PFGE), amplified fragment length polymorphism (AFLP), fla short variable region (fla-SVR), fla restriction fragment length polymorphism (fla-RFLP), multi-locus sequence typing (MLST), microarray comparative genomic hybridization (MCGH), major outer membrane protein (MOMP), whole genome sequencing (WGS).

Typing methods should incorporate versatility by providing high discriminatory power to identify the isolates relatedness in order to link the causative agent with the outcome either for foodborne outbreaks detection or longitudinal surveillance [76]. In this section we will focus on the most widely used molecular typing techniques that are at the forefront of current research (Figure 1). Molecular methods based on DNA electrophoresis or single loci analysis, including pulsed-field gel electrophoresis (PFGE) fingerprinting, restriction fragment length polymorphism (RFLP) analysis and *flaA* short variable region (SVR) typing, as well as the multi-locus sequence typing (MLST), the major outer membrane protein (MOMP) schemes and the whole-genome sequencing (WGS) have provided significant insights into the similarities among *Campylobacter* isolates stemming from human disease and environmental reservoirs such as farm animals and water [77,78].

PFGE is the first DNA-based typing method applied for *Campylobacter* spp. and is generally considered as the “gold standard” technique for the typing of a multitude of important pathogens [79,80]. With a total of 283 hits in a PubMed search using the query (“PFGE” and “*Campylobacter*”), the PFGE method has been the most widely adopted epidemiological tool from 1991 to present. The success of PFGE stems from the high discriminatory power it offers in both outbreak investigation and epidemiological surveillance. PFGE has remained the primary molecular typing method for almost for three decades, and is a cost-effective tool with high reproducibility among different laboratories. Bioinformatics, in accordance with the standardized protocols of PFGE, have offered substantial help in a worldwide fingerprinting of *Campylobacter* and other foodborne isolates as well as for the monitoring of emerging clones; this method actually set the basis for the implementation of PulseNet in the U.S. [81]. Despite the significant advantages of PFGE, the increased difficulty in workload, the lack of rapidity in analysis, the difficulty in intralaboratory communication of the results, as well as the low resolution of the method in distinguishing bands of relatively same size has set several serious limitations [73,75]. The amplified fragment length polymorphism (AFLP) typing method offers high discriminatory power, portability of the analysis results and high reproducibility, but the costly equipment and the difficulties in its use set serious limitations [73,75]. These limitations along with the fact that it is a more recent method than PFGE may partially explain the reduced application frequency.

Studying bacterial isolates relatedness based on a single target gene is the field of the single locus sequence typing (SLST) method. For *Campylobacter,* nucleotide sequencing of a short variable region (SVR) of a gene provides significant information for the *Campylobacter* “fingerprint” [75,76,82]. The widely applied sequencing of the SVR of the flagellin A (*flaA*) and flagellin B (*flaB*) genes is a simple, rapid and low-cost method with high discriminatory power that supersedes the previously performed flagellin-based restriction fragment length polymorphism analysis (*fla*-RFLP) for *Campylobacter* isolates discrimination [76,83,84,85]. The potential low-reliability of the SLST methods for *Campylobacter* spp. lies in the highly variable genome of the isolates due to the naturally occurring genetic elements uptake, recombination and alleles instability [86,87]. Therefore, questions have been raised whether SLST methods are appropriate for long-term and large-scale investigation of closely related *Campylobacter* strains.

The first *Campylobacter* MLST scheme was developed to discriminate between *C. jejuni* and *C. coli*; this scheme required the sequencing of seven stable housekeeping genes (*asp, glnA, gltA, glyA, pgm, uncA, tkt*) [88,89]. For other *Campylobacter* spp. the MLST with variant (rMLST) scheme presented slightly different substitutions of several genetic loci among the various species [90,91]. Although a rather new technique, MLST has gained attention due to the excellent reproducibility when used in epidemiological studies on a large-scale and in genetic studies of the *Campylobacter* population. An interesting notification that needs to be addressed is the numerical advantage of MLST publications over PFGE, despite the fact that the latter is the first typing method applied for *Campylobacter* spp. and is widely accepted to be a “gold-standard” typing technique. MLST seems to have been more widely and rapidly adopted during the last decade (Figure 1). More specifically, MLST data applied for the genetic structure of the *Campylobacter* population have improved our understanding on the various routes of transmission leading to human disease. These data are electronically portable and sequence type profiles are available online in two central databases (http://pubmlst.org and www.mlst.net), while the eBURST online software is used in the determination of bacterial genetic relatedness and clinical relevance, offering a valuable tool in designing prevention strategies to promote the reduction of human campylobacteriosis and its sequelae [75,92,93,94]. Additionally, the ability of the widely studied *C. jejuni* to adapt in adverse environments has led to genetic instability and phenotypic diversity, thus enhancing the survival of the species and this characteristic can be studied by the MLST method [95,96]. Albeit its contribution for a deeper insight into the structure, evolution, sequence diversity and genetic instability of the *Campylobacter* population, MLST presents limitations in its implementation in outbreaks due to the high-cost and time-consuming analysis [76].

Further characterization of the isolates based on gene encoding for the bacterial outer membrane protein content is known to improve *Campylobacter* epidemiological identification through genetic discrimination [97]. Specifically, for *C. jejuni* and *C. coli*, the presence of the the *porA* gene encoding for the MOMP porin A has been demonstrated, while for *C. fetus* the genes *cmp1* and *cmp2* encode for two porin-like activity MOMPs other than porin A [97,98]*.* Regional outbreak investigations regarding foodborne human campylobacteriosis underline the usefulness of MOMP typing to triage environmental *Campylobacter* isolates before conducting more laborious molecular typing analysis [99]. The MOMP typing method is very recent and is mostly used for *C. jejuni* typing. To date, the *cmp*-based typing method is considered a tool with high discriminatory power which is simple in use [98,100].

DNA microarrays using probes that are complementary to specific bacterial nucleotide sequences, represent a rapid method for detecting genes or alleles of particular bacterial species in a single experiment [75,76]. Extensively used in *C. jejuni* research studies, microarray comparative genomic hybridization (MCGH) has yielded successful genomic analysis of the highly variable genome of this pathogen. However, microarrays are ultimately abandoned and cannot be considered as optimal tools for subtyping because they offer limited throughput in real-time outbreak investigation and constitute a costly and a technique that is difficult to standardize. Another method of comparative genomics is the recent typing method of comparative genomic fingerprinting (CGF), which has improved routine campylobacteriosis surveillance [101]. The CGF is the preferred method for the detection of *Campylobacter* genes with high variability among the different species of bacterial clusters that have been previously identified by the MCGH method [102].

Finally, the revolutionary next generation sequencing (NGS) methods promise a broader application of high-resolution bacterial WGS, which, however, remains a laborious tool in the daily routine of research and clinical laboratories. Genome data deriving from WGS will soon comprise useful information for detection and evaluation of the bacterial pathogens that are close phylogenetic neighbors and play an important role in public health [75,103]. In the era of metagenomic sequencing-based tools, public health microbiology is undergoing substantial changes; WGS replaces traditional phenotypic tests and narrowed-spectrum genetic methods that apply to universal or species-specific markers with low discriminatory power for subtyping analyses. The application of high quality WGS provides the research community a wider array of *Campylobacter* reference genomes for exploitation. In fact, WGS enables the analysis of multiple strains within a bacterial sample and offers a comprehensive genome sequence data set, a property previously unavailable given that routine subtyping methods provided restricted discriminatory power in non-clonal populations with high genetic diversity [104]. WGS will allow the detection of epidemiological variations among strains and will progressively replace traditional typing methods, but before this, issues related to genetic diversity have to be addressed in order to define the cut off values and criteria that determine which infections stem from clonal isolates or a common source [56,105].

In general, molecular typing and automated sequencing techniques have led to significant accomplishments in diagnostics and biotechnology. NGS technologies offer subtyping data to improve programs based on risk-based sampling algorithms with rapidity, simplicity and at a low cost. Platforms based on sequencing technologies are progressively reported in the monitoring of poultry production aiming to improve food safety in the market and consequently public health [106]. Additionally, NGS technologies have improved our understanding of the bacterial methylation processthat greatly affects pathogenicity. Such an example for the use of NGS in campylobacters is the application of single molecule real-time (SMRT) sequencing for the detection of methylation patterns in *C. jejuni* [107,108]. Lastly, the notion that NGS offers DNA characterization at large and complex populations paves the way towards an improved experimental procedure in the characterization of multispecies and multi-subspecies populations [109].

It is a fact that the majority of research has focused on evaluating the molecular typing and subtyping methods applied for *C. jejuni* and *C. coli*, which are two genetically diverse species due to their innate property of extracellular DNA uptake from horizontal genetic exchange [110]. The highly variable genome of these *Campylobacter* species has raised concerns regarding the application of typing methodologies based on DNA sequence information such as the MLST, *flaA* SVR, and the MOMP [111,112]. This obstacle paved the way towards combining methodologies for a more accurate *Campylobacter* populations investigation, such as Multiplex Ligation-Dependent Probe Amplification–Binary Typing [102,113]. Genomic mosaicism can adversely impact the understanding of closely related species and the use of few genetic loci can lead to erroneous results. A promising combination could be the MLST method applied with MCGH offering high discriminatory power even in *Campylobacter* populations with extensive recombination and genomic mosaicism [114].

Another burden in typing is the fact that several molecular techniques, such as the random amplification of polymorphic DNA (RAPD), the arbitrarily primed polymerase chain reaction (AP-PCR), the repetitive-element polymerase chain reaction (rep-PCR), and the variable-number tandem repeat typing (VNTR), can be applied for epidemiological surveillance only at a local level, and the data deriving from the analysis cannot be interchangeable; specifically, for *Campylobacter* typing only a few reports exist for these methodologies [73,75]. Such typing tools possess poor reproducibility and low discriminatory power, despite their rapidity, ease of use and low cost.

## 4. Conclusions

The engagement of the state-of-the-art typing methods with human campylobacteriosis and environmental reservoirs is the main focus of the study of *C. jejuni* and *C. coli*. Our limited ability to understand the interaction between *Campylobacter* and the host lies in the absence of a systematic effort to monitor, evaluate and compare this bidirectional relationship. Additionally, the absence of a “gold standard” identification method—which is mainly attributed to the high variability and genetic instability of *Campylobacter* spp.—restricts the actual range of isolates. This characteristic inevitably makes laboratory diagnosis difficult, since culture-dependent methodologies are effective only for a small subset of *Campylobacter* species. Considering how many of the *Campylobacter* strains isolated globally have gone under-determined, the idea of an efficient and approved storage and maintenance protocol could significantly offer a great advantage in the study of more *Campylobacter* species by currently applied isolation and typing methods. Moreover, in terms of subtyping, there is the need for the development of assays with the ability to target genetic variation so that we can differentiate the various lineages in the population for epidemiological tracking. The fact that there is not a “gold standard” subtyping methodology is a reflection of the insufficient data on *Campylobacter* population genetics due to the absence in tools for detection/identification either at the microbiological or molecular level.

Polyphasic studies for strain taxonomic position classification involving universally applicable molecular methodologies and enable accurate and safe results at a species and subspecies level, such as MALDI-TOF MS and WGS, could offer a high degree of agreement on epidemiologic surveillance. Reliable molecular typing along with the subsequent phylogenetic analysis of *Campylobacter* strains isolated from both clinical settings and the broader environment would contribute to the epidemiological database indispensable for every laboratory. This database should ideally include all the phenotypic and genotypic features, the demographic and clinical data of the patients when it comes to clinical human isolates as well as information about the natural reservoirs of the *Campylobacter* spp. in both animals and water. Such an accessible and comparable intralaboratory database would significantly enhance epidemiological surveillance on a local and universal scale, and would function as a tool for the control of campylobacteriosis. A consistent surveillance system means more than epidemiological surveillance at a national level; it means (i) better understanding, (ii) closer monitoring, (iii) targeted action design, and (iv) efficient strategy implementation.
